# Gum Guar fiber associated with fructose reduces serum triacylglycerol but did not improve the glucose tolerance in rats

**DOI:** 10.1186/1758-5996-2-61

**Published:** 2010-10-27

**Authors:** Caio SM Motoyama, Mônica JS Pinto, Fabio S Lira, Eliane B Ribeiro, Claudia MO do Nascimento, Lila M Oyama

**Affiliations:** 1 Departamento de Fisiologia. Universidade Federal de São Paulo - UNIFESP - Rua Botucatu, 862, 2nd floor, Edifício de Ciências Biomédicas. Vila Clementino, São Paulo, SP, Brazil

## Abstract

**Introduction:**

The increased intake of dietary fructose can be associated with alterations on energy homeostasis and lipid/carbohydrate metabolism, such as insulin resistance and dislipidemia. On the other hand, the ingestion of soluble fiber gum guar could improve benefic mechanism on glucose tolerance and lipids profile.

**Objective:**

The aim of the present study were to investigate the effects of the supplemental feeding partially hydrolyzed gum guar on glucose and lipid homeostasis, in rats fed with fructose solution.

**Methods:**

The study was performed on thirty day-old male Wistar rats randomly assigned into four groups: control(C) or treated with fructose (F-20%), fiber (FB-5%), or fructose plus fiber (F-20% + FB-5% = FF) solution for 30 days on glucose tolerance (OGTT), triacylglycerol concentration in the liver by chloroform/methanol method, glucose, triacylglycerol and total cholesterol serum concentration by assayed by enzymatic colorimetric method, insulin receptor (IR) concentration in the liver by Western Blotting.

**Results:**

The total body weight gain was not different between groups; in regards of total caloric intake, in the F group was significantly higher and in the FB group was lower than other groups. The triacylglycerol concentration in the liver of FF group was significantly higher than F group, the triacylglycerol concentration in the serum was higher the F group compared with other groups. The OGTT reveal impaired on glucose tolerance in the F, FB, FF compared with C. The IR concentration in the liver was lower in the F, FB, FF compared with C, no significant difference was observed between groups for IR concentration in the gastrocnemius muscle. No significant difference was observed between groups for carcass fat content and serum total cholesterol.

**Conclusion:**

Fructose induced important alterations on glucose tolerance and lipid metabolism, despite of fiber showed reversion of part this alterations. The association fructose plus fiber to seem decrease insulin receptor concentration in the liver, with consequent impair on glucose tolerance.

## Introduction

Insulin resistance is a key feature of impaired glucose tolerance in type 2-diabetes that can be characterized by a diminished ability of insulin sensitive tissues and a marked decrease of glucose metabolism in response to insulin. The dyslipidemia associated with insulin-resistant states is characterized by hypertriacylglycerolemia, an increase in hepatic VLDL secretion, and a decrease in peripheral triacylglycerol clearance [1].

The development of insulin resistance can be linked to both genetic and environmental factors [2,3]. One of the most likely environment factors is the habitual diet. Ingestion a high fructose provides a dietary model of insulin resistance associated with hyperinsulinemia and low glucose uptake by peripherics tissues [4-7], hypertriacylglycerolemia [8-11] and hypertension [5,12].

Fructose consumption markedly increases circulating postprandial triacylglycerol concentrations [4,13] by increasing hepatic de-novo lipogenesis, which in turn up regulates VLDL production and secretion [10], since the liver is the main site of fructose metabolism [14]. In addition, it has been suggested that hepatic triacylglycerol accumulation is a major mediator of hepatic insulin resistance, although there is also contradictory evidence [2].

Nevertheless, feeding fibers, such as guar gum fiber, a no digestible saccharide with a high viscosity, provides benefits associated with modifies glucose tolerance, lowers plasma triacylglycerol in rats [15-18], by promote modified on carbohydrates absorption in the bowel. Suzuki and Hara [4] showed that the addition of gum guar hydrolysates to the fructose diets lowered triacylglycerol levels in liver, as well as in plasma.

However, the mechanism of the effects of low-viscosity gum guar hydrolysates on glucose tolerance and insulin resistance, which is associated with dyslipidemia, has not been investigated totality.

The aim of the present study were to investigate the effects of the supplemental feeding partially hydrolyzed gum guar on glucose and lipid homeostasis, in rats fed with fructose solution.

## Materials and methods

### Animals and treatments

Male Wistar Rats aged 30 days and weighting 40-50 g, were obtained from Sao Paulo Federal University Experimental Models Development Center (CEDEME). The rats were housed inside standard polypropylene cages in a temperature-controlled (24 ± 1°C) room with a 12:12 h light-dark cycle (light on at 07:00 hours). This study was approved by Sao Paulo Federal University Research Ethics Committee (0433/07). Food, water or solutions content fructose, fiber and fructose plus fiber were provided *ad libitum *by placing chow pellets and solutions bottles on a grid located on top of the chamber.

The animals were randomly distributed in 4 different groups were fed on a regular rodent chow (PURINA 502): home-cage control with water normal (C n = 12), home-cage fructose with 20% of drinking water was composed of fructose solution (F n = 12), home-cage fiber with 5% of drinking water was composed of partially hydrolyzed Guar Gum fiber solution (FB n = 12) and home-cage fructose plus fiber with 20% of drinking water was composed of fructose solution plus 5% of drinking water was composed of partially hydrolyzed Guar Gum (FF n = 12).

### Protocols of study

#### Oral Glucose Tolerance Test (OGTT)

All animals were left 12 hours fast. Initially, the baseline blood was collected to assess basal glucose concentration, from the tail vein. Then the glucose solution Merck^® ^(2 g/kg of body weight) was administrated by intragastric gavage. Blood samples were collected at 15, 30, 45, 60 and 90 minutes to measure glucose concentration.

After the last tail blood collection, the animals were sacrificed. Trunk blood samples were collected and centrifuged (2500 rpm X for 30 min at 4°C) and serum was separated and storage - 80°C for measure concentrations of triacylglycerols and total cholesterol.

The liver were removed and frozen in appropriate protein extraction buffer.

#### Method of lipids hepatic extraction

The liver was eviscerated, weighed, and stored at -20°C. Triacylglycerol content in the liver was measured as described by Folch et al. [19].

#### Western Blot Analysis

Insulin receptor from liver was quantified by Western Blotting (WB). The liver was removed and the tissues were homogenized in 0.9 mL of protein extraction buffer (1% Triton X-100, 100 mM Tris-HCl (pH 7.4), 100 mM sodium pyrophosphate, 100 mM sodium fluoride, 10 mM EDTA, 10 mM sodium orthovanadate, 2.0 mM phenylmethylsulfonyl fluoride, and 0.1 mg aprotinin/mL). Total protein concentration was determined by Bradford method (1976) [20], using albumin as standard. The respective liver homogenates (120 μg of protein for IR) was separated on an SDS 8% polyacrylamide gel. The protein were electrophoretically transferred to a nitrocelulose membrane (GE Health Care), and then blocked with 1% albumin solution for overnight at 4°C.

The membrane was incubated with the monoclonal insulin receptor (IR) antibody (Cell Signaling Technologies) (1:10.000) for 2 h at room temperature. After three washes, the membrane was incubated with a peroxidase conjugated secondary antibody (Santa Cruz Biotechnology) (1: 500) for 1 h at room temperature. After three additional washes, immunoreactive bands were detected using the Enhanced Chemiluminescence Assay System Plus (GE Health Care). The same membrane was stripped and reblotted with α-tubulin antibody.

Autoradiograms then underwent semiquantitative densitometric analysis. The data were expressed as means ± SEM of arbitrary units in relation to those of the correspondence α-tubulin bands. The optical density of the immunoreactive bands was calculated by using Image J software.

#### Biochemical serum analysis

Serum glucose, triacylglycerols, total cholesterol were assayed by enzymatic colorimetric method using commercially available kits (Labtest^®^).

#### Statistical Analysis

All results are presented as means ± standard error of the mean (SEM). Statistical significances were assessed using two-way analysis of variance (ANOVA) followed by Tukey test *as a post hoc *analysis to identify significant differences among the groups. Differences were considered significant when p < 0.05.

## Results

### Body weight and food intake

**Figure 1:**
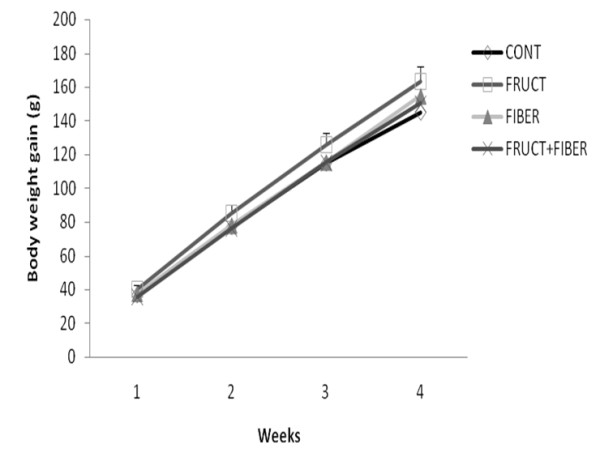
**Total body weight gain of rats control or treated with fructose, fiber and fructose plus fiber during 4 weeks**. Data are expressed as mean ± SEM. n = 12.

**Figure 2:**
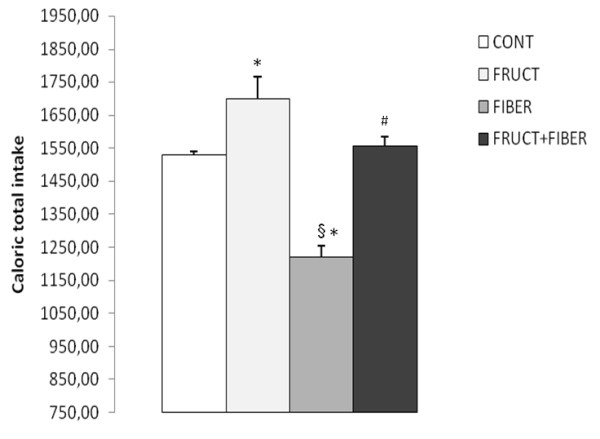
**Total caloric ingestion of rats control or treated with fructose, fiber and fructose plus fiber during 4 weeks**. Data are expressed as mean ± SEM. n = 12. * p < 0.05 Different from control group, §p < 0.05 Different from fructose group, # p < 0.05 Different from fiber group.

Body weight and food intake were monitored in all groups of rats throughout the whole experiment period. During the treatment period, the total body weight gain was not different between all studied groups (Figure 1). In regards to total caloric intake, in the F group was significantly higher, however, in the FB group it was significantly lower than other groups. No significant difference was observed between C and FF group (Figure 2).

### Liver and serum triacylglycerol concentration

**Figure 3:**
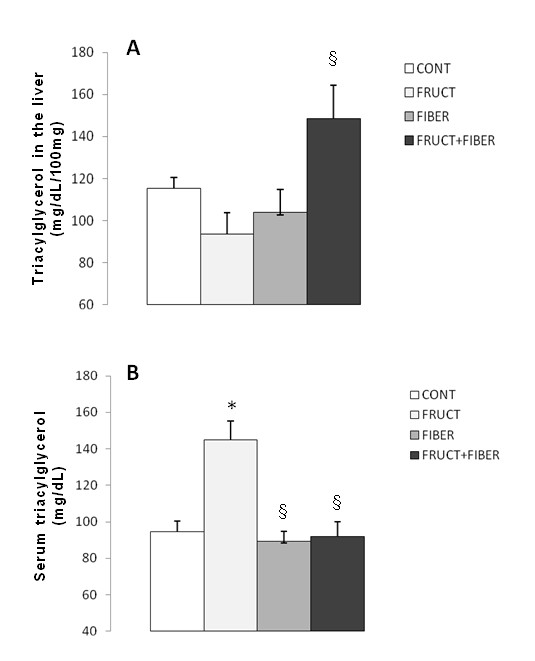
**The triacylglycerol concentration in the liver (panel A) and serum (panel B) of rats control or treated with fructose, fiber and fructose plus fiber in during 4 weeks**. Data are expressed as mean ± SEM. n = 4-12. *p < 0.05 Different from control group, § p < 0.05 Different from fructose group.

The triacylglycerol concentration in the liver of FF group was significantly higher than F group (Figure 3A). Depicts triacylglycerol concentration in the serum of all groups, ANOVA revealed a significant increase in the F group compared with other groups (Figure 3B).

### Serum total cholesterol concentration

**Figure 4:**
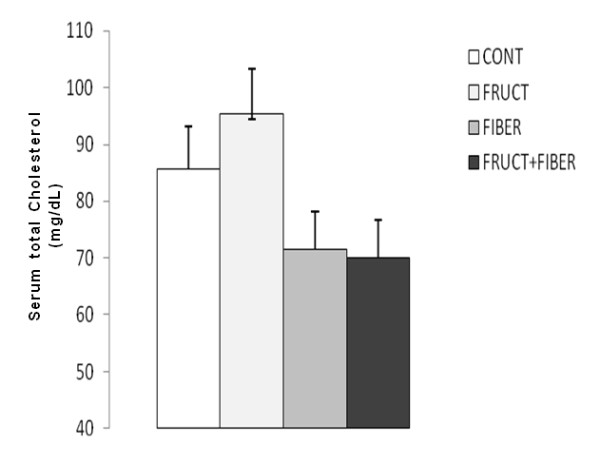
**Serum total cholesterol concentration of rats control or treated with fructose, fiber and fructose plus fiber in during 4 weeks**. Data are expressed as mean ± SEM. n = 5.

No significant alteration in the serum total cholesterol (Figure 4) was observed in the four groups evaluated.

### Quantification of protein expression of insulin receptor (IR) in the liver

**Figure 5:**
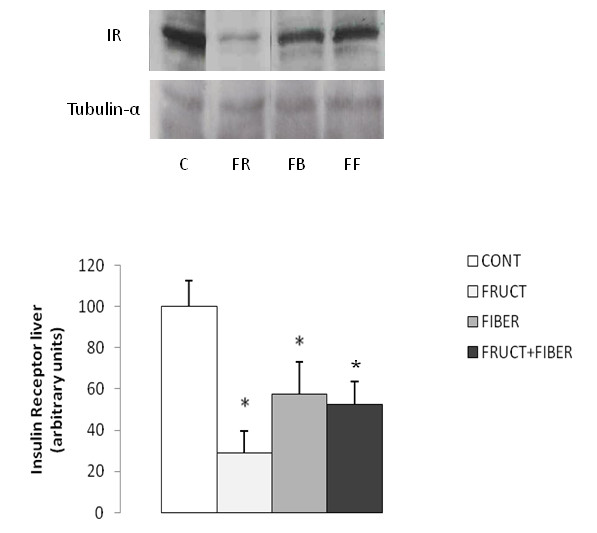
**Insulin receptor (IR) concentration in the liver of rats control or treated with fructose, fiber and fructose plus fiber in during 4 weeks**. Data are expressed as mean ± SEM. n = 4. * p < 0.05 Different from control group.

The Western Blotting experiment showed that IR content decreased in liver in the F, FB and FF group compared C group (Figure 5).

### Oral glucose tolerance test (OGTT)

During baseline the average fasting serum glucose concentration was higher FF group compared all other groups. After 15 minutes, was demonstrated that the glucose concentration in the FF group was significantly higher compared with F and C, remaining higher compared C group in the 30 minutes.

The glucose in the F group was higher in the 30 minutes compared with C and FB groups, remaining higher compared C group after 45 and 60 minutes of glucose administration.

The oral test revealed that the glucose was significantly higher in FB in the 30 and 45 minutes compared with C group.

**Figure 6:**
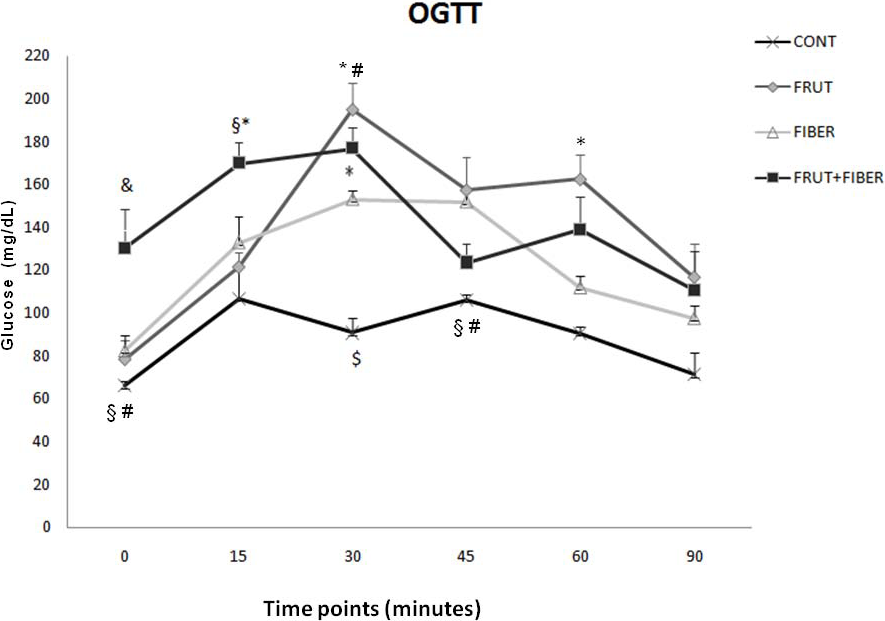
**Oral glucose tolerance test (OGTT) of rats control or treated with fructose, fiber and fructose plus fiber in during 4 weeks**. Data are expressed as mean ± SEM. n = 4.* p < 0.05 Different from control group. # p < 0.05 Different from fiber group,§ p < 0.05 Different from fructose group,$ p < 0.05 Different from fructose plus fiber group.

No significant difference was observed between all groups in the 90 minutes (Figure 6).

## Discussion

The consumption of fructose has increased considerable during the last several years and it is related, partially, for the increasing of the obesity and metabolic syndrome, since fructose promotes hypertriacylglycerolemia, hyperglycemia, hypertension [5-12].

The body weight gain was not different among all experimental groups, although the high caloric intake from the fructose animals and low, from fiber group as compared to the control group. These results are in accordance with other, which demonstrated that fructose treatment did not change the body weight [21-23].

Even the different treatments made alteration on caloric intake, they change the trialcilglycerol levels, both serum and liver concentration, as the distinct pattern.

The consumption of fiber did not change the serum and liver triacylglycerol levels as compared to control group. Fernandez et al. [24] demonstrated low serum triacylglycerol level but not change in liver VLDL secretion when guinea-pig was fed with 12.5% guar gum.

On the other hand, fructose treatment promoted an increase in serum triacylglycerol level. This result is in accordance with other, showing differences related to the time of treatment and concentration of fructose. Catena et al. [5] developed a total carbohydrate substitution by fructose for 2 week, while Jürgens et al. [25] and Girard et al. [21] treated with a solution (15% and 18% respectively) for 73 days or 8 weeks, respectively. These results demonstrated that although the time of treatment and the concentration of fructose used in our protocol were lower than given from the literature, it was efficient to promote alteration on serum lipid profile. Chong et al. [26] demonstrated that a high ingestion of fructose decrease the lipase lipoprotein enzyme activity which lead a low triacylglycerol clearance.

Interestingly, the association of fructose with fiber normalized the triacylglycerol serum level and also, increased the triacylglycerol concentration on liver. It can be suggested that this association could promote a reduction on VLDL secretion from liver.

Basciano et al. [10] shown that high dose of fructose intake promoted hypercholesterolemia. Even though it did not mean a statistically difference, we observed a tendency to increase the cholesterol level in fructose group as compared to control one. Perhaps a longer treatment could promote a significant difference between fructose and control groups.

Hypertriacylglycerolemia is associated with insulin resistance development [2]. Treatment with fructose, fiber or association between both nutrition factor shown a lower liver insulin receptor concentration than control one. Previously, some relate demonstrated hepatic insulin resistance associated with a high fructose intake [2,27], independent of changes in body composition, and serum free fat acid and leptin concentration [28].

There was no change in liver triacylglycerol concentration from fructose group; consequently the reduction on liver insulin receptor could not be related to the change in liver triacylglycerol level

Increase in liver fat deposition is correlated with insulin resistance [29-31]. On contrary some authors demonstrated that liver insulin resistance could occur dissociated with liver fat accumulation [32-34]. Also, others observed hepatic insulin resistance associated with triacylglycerol serum concentration [35-37], similar with the fructose treated animals in our protocol. Hence, it is suggested that the decrease in liver insulin receptor could be related to the increase in serum triacylglycerol level.

The association of fructose with fiber was not efficient to return the liver insulin receptor to normal level. A higher flux of fructose provenient originating of diet is capable of induce increase of lipogenesis *de novo *in the liver, resulting accumulate triacylglycerol in the liver, with consequent higher production of VLDL and insulin resistance [31]. In agreement, the triacylglycerol concentration in the liver of FF group was significant higher than F group, what can suggest insulin resistance.

The consumption of fiber one-off promoted also a decrease in liver insulin receptor. It seems a paradox, but it is known that guar gum decrease insulin secretion by delaying the glucose absorption from gut and its low glycemic index [17]. Probably, the low requirement for insulin secretion could promote a down regulation of insulin receptor in the liver. These indicate that the hepatic insulin sensibility is affected by fructose and/or fiber, through different mechanisms. Others studies are necessary to better understand the mechanism involved in this process, specially the quantification of insulin signally.

In fact, the oral glucose tolerance test showed an alteration in all experimental groups which presented low liver insulin receptor. It is well demonstrated that glucose uptake and metabolism are depend on gut absorption rate, insulin secretion, glucose liver utilization and metabolism, and glucose uptake from tissues like muscle and adipose tissue [38].

In conclusion, the fructose intake promoted a lipidic and glycidic metabolic imbalance. The association of fructose with fiber revert partially this imbalance, especially concerning on triacylglycerolemia. However, the administration of fructose and/or fiber produced a decrease in liver insulin receptor, triggering glucose intolerance.

## Competing interests

The authors declare that they have no competing interests.

## Authors' contributions

CSMM - participated in the design of the study, and carried out all the experimental procedures. MJSP - carried out the Western blotting and OGTT procedure. FSL - drafted the manuscript, performed the statistical analysis. EBR - drafted the manuscript, performed the statistical analysis. CMON - participated in the design of the study, performed the statistical analysis. LMO - participated in the design of the study, and coordination. All authors read and approved the final manuscript.
